# Progress of Research in Negative Thermal Expansion Materials: Paradigm Shift in the Control of Thermal Expansion

**DOI:** 10.3389/fchem.2018.00267

**Published:** 2018-07-02

**Authors:** Koshi Takenaka

**Affiliations:** Department of Applied Physics, Nagoya University, Nagoya, Japan

**Keywords:** negative thermal expansion, phase transition, thermal expansion control, composites, microstructural effects

## Abstract

To meet strong demands for the control of thermal expansion necessary because of the advanced development of industrial technology, widely various giant negative thermal expansion (NTE) materials have been developed during the last decade. Discovery of large isotropic NTE in ZrW_2_O_8_ has greatly advanced research on NTE deriving from its characteristic crystal structure, which is now classified as *conventional* NTE. Materials classified in this category have increased rapidly. In addition to development of conventional NTE materials, remarkable progress has been made in *phase-transition-type* NTE materials using a phase transition accompanied by volume contraction upon heating. These giant NTE materials have brought a paradigm shift in the control of thermal expansion. This report classifies and reviews mechanisms and materials of NTE to suggest means of improving their functionality and of developing new materials. A subsequent summary presents some recent activities related to how these giant NTE materials are used as practical thermal expansion compensators, with some examples of composites containing these NTE materials.

## Introduction

Control of thermal expansion, an urgent demand in modern industrial technology, is making remarkable progress. Even a minute change of 10^−5^ in linear distortion fatally degrades the performance of high-precision devices and instruments. Furthermore, for a device comprising multiple materials, mismatched thermal expansion between the constituents themselves can cause severe damage such as exfoliation of interfaces and breakage of wires. In addition to work in fields that have traditionally avoided the adverse influence of thermal expansion such as optical instruments and precise machining equipment, control of thermal expansion has been strongly sought in recent years for advanced electronics such as power semiconductor devices, thermoelectric conversion systems, and fuel cells. Difficulties attributable to thermal expansion control are universal and are difficult to resolve.

**Figure 1:**
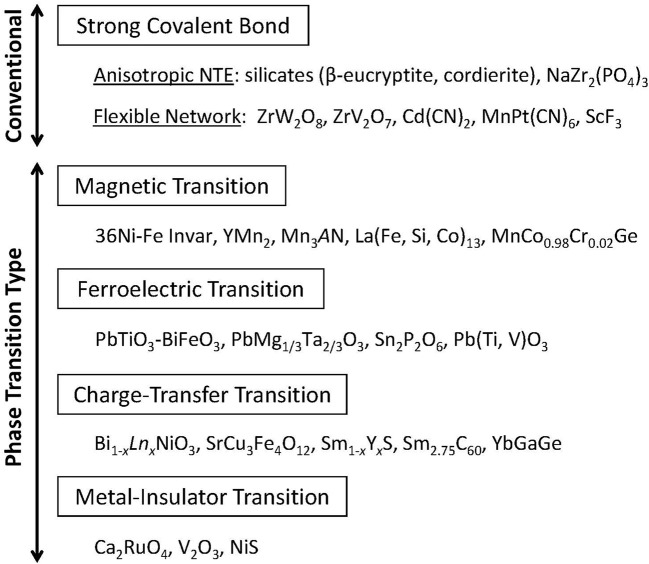
Classification of negative thermal expansion materials. Materials are divisible into two categories: *conventional* and *phase-transition-type* materials.

The core technology of thermal expansion control is *negative* thermal expansion (NTE) materials: substances that contract when heated (Chu et al., 1987; Sleight, 1998; Evans, 1999; Barrera et al., 2005; Lind, 2012; Takenaka, 2012; Chen et al., 2015). After discovery of the large isotropic NTE of ZrW_2_O_8_ in 1996, for which the coefficient of linear thermal expansion α_L_ (subscript L signifying “linear”) reaches −9 × 10^−6^ K^−1^ (Mary et al., 1996), the successive discovery of giant NTE materials has encouraged remarkable progress of research in this field, particularly during the last decade. Such development has brought a paradigm shift for the control of thermal expansion. For further improvement of the NTE functions and for the development of new materials, the achievements of the last two decades in this field are summarized briefly, particularly addressing recently discovered giant NTE materials (Figure 1).

## Positive and negative thermal expansion

### Thermal expansion of solids

The origin of thermal expansion of solids can be summarized briefly (Cochran, 1973). One might consider atoms connected together by springs as a model of solids, but these springs are *anharmonic* and do not exactly and simply obey Hooke's law. The atoms are prevented from becoming extremely close to one another because of Pauli's exclusion principle. As a result, excursions to longer interatomic distances occur more readily than those to shorter interatomic distances. Consequently, the average interatomic distance increases concomitantly with increasing temperature *T*. More intuitively, one can generally infer from Pauli's exclusion principle that as the temperature rises and the thermal vibration of an atom increases, it tries to maintain its distance from other atoms to avoid mutual collision. This increasing distance with vibration explains thermal expansion.

### Conventional NTE materials

**Figure 2:**
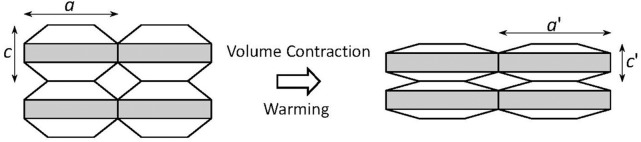
Schematic of anisotropic thermal expansion in the silicates. As the shaded layers undergo thermal expansion, they are pulled closer together in the direction perpendicular to the layer, which causes significant thermal contraction in this direction and yields slight net volumetric thermal contraction.

In stark contrast to ordinary materials, some materials contract upon heating. Some examples are those of a silicon oxide family including β-eucryptite (LiAlSiO_4_) and cordierite (Mg_2_Al_2_Si_5_O_18_), which were reported in the 1950s (Gillery and Bush, 1959). These silicates have strong covalent bonds such as Al–O and Si–O bonds, and not so strong ionic bonds such as Li–O and Mg–O bonds. The ionic bonds expand on heating, resulting in the expansion of two-dimensional sheets in β-eucryptite, for example. Thermal expansion of these ionic bonds pulls the two-dimensional sheets closer together because these two-dimensional sheets are joined by strong covalent bonds that do not expand when heated (Figure 2). For β-eucryptite with a hexagonal crystal lattice, on warming from 293 K to 1073 K, the in-plane (*a* axis) direction in which the Li–O bond is dominant expands 0.62%, whereas the out-of-plane (*c* axis) direction, in which the Si–O bond is dominant, contracts 1.39%. This thermal distortion causes unit-cell volume contraction of 0.15%. From another viewpoint, open spaces are filled by the thermal distortion of strong covalent bonds. This concept is exemplified more clearly in the open-framework or flexible-network materials described next. Readers might doubt that the NTE of β-eucryptite is much larger. Details of the discrepancy between the unit-cell volumetric NTE and the bulk NTE are discussed in Chapter 4.

**Figure 3:**
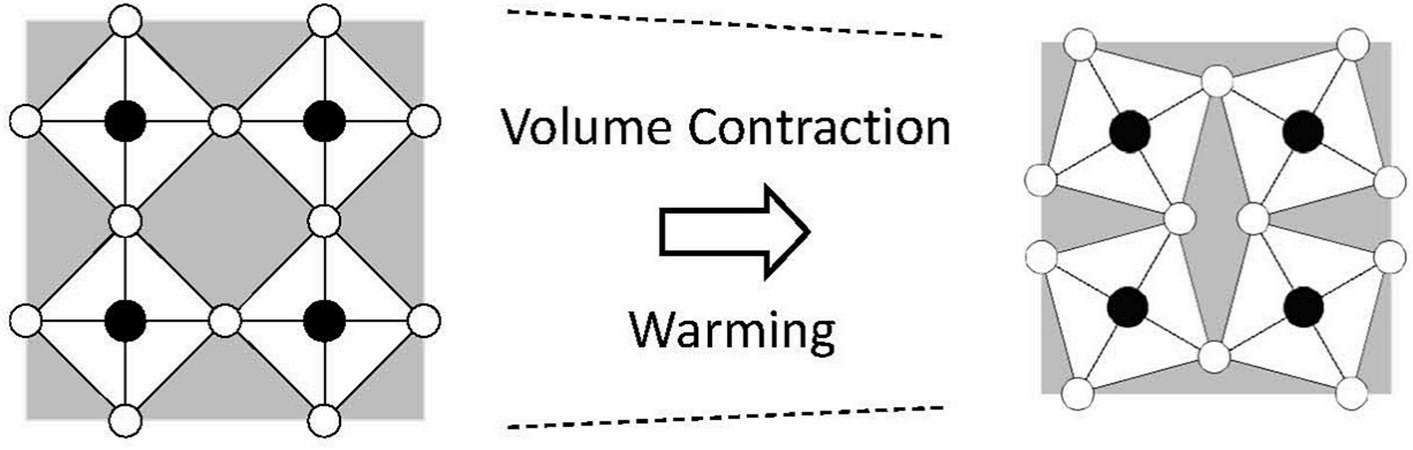
Schematic of negative thermal expansion in a flexible network. A vibrational mode consuming an open space in a crystal lattice is thermally excited, which yields net volumetric thermal contraction.

Flexible-network materials are a well-known family of NTE materials that includes ZrW_2_O_8_ and vanadates and phosphates of certain kinds (Mary et al., 1996; Pryde et al., 1996; Evans et al., 1997). This network consists of rigid units connected by soft linkages. The rigid units are formed by strong covalent bonds. For ZrW_2_O_8_, W–O covalent bonds are strong. Therefore WO_4_ units are rigid. They do not expand on heating. By contrast, although the W–O and Zr–O bond distances are not changed, the Zr–O–W linkages are soft. Transverse oxygen displacement is induced easily on heating. These displacements consume open spaces in the crystal structure, resulting in volumetric NTE (Figure 3). Different from the silicates, anisotropic lattice thermal expansion is unimportant. The volume change related to NTE is much greater. In fact, ZrW_2_O_8_ has attracted great attention because of its large and isotropic NTE of α_L_ = −9 × 10^−6^ K^−1^ (Mary et al., 1996). NTE appears at the whole *T* range below 1443 K. The total volume change related to NTE, Δ*V*/*V*, reaches 2.7% (Evans, 1999).

Negative thermal expansion that is explainable from the concept that strong atomic bonds and thermally induced deformation fill open spaces in the crystal lattice explains *conventional* NTE materials (Figure 1), which include widely diverse materials. In addition to cyanides (Chapman et al., 2006; Phillips et al., 2008), the fluoride group (Greve et al., 2010; Attfield, 2011; Chatterji et al., 2011; Chen et al., 2017), such as ScF_3_ and ZnF_2_, has attracted attention recently. Particularly, Cd(CN)_2_·*x*CCl_4_ shows large NTE of α_L_ = −34 × 10^−6^ K^−1^ and Δ*V*/*V* = 2.1% at *T* = 175–375 K (Phillips et al., 2008).

At present, most industrial thermal-expansion compensators, including β-eucryptite, belong to the group of conventional type oxides. This type of NTE has a structural origin for its phenomena. Therefore, NTE appears in almost the entire *T* range, which is extremely important industrially. However, when discussing practically used materials, their NTE magnitude, at most α_L_ = −7 × 10^−6^ K^−1^, is not large from today's perspective. Because the coexistence of strong and not strong chemical bonds is fundamentally important for conventional NTE, low thermal conductivity and low stiffness are unavoidable in these materials. Low thermal conductivity and low stiffness are weak points even for some *phase-transition-type* NTE materials described in the next chapter. Diligent efforts are continuing toward overcoming them. For example, high thermal conductivity is indispensable for rapid heat dissipation of devices. Development of NTE materials with high thermal conductivity is desired.

## Phase-transition type NTE materials

**Figure 4:**
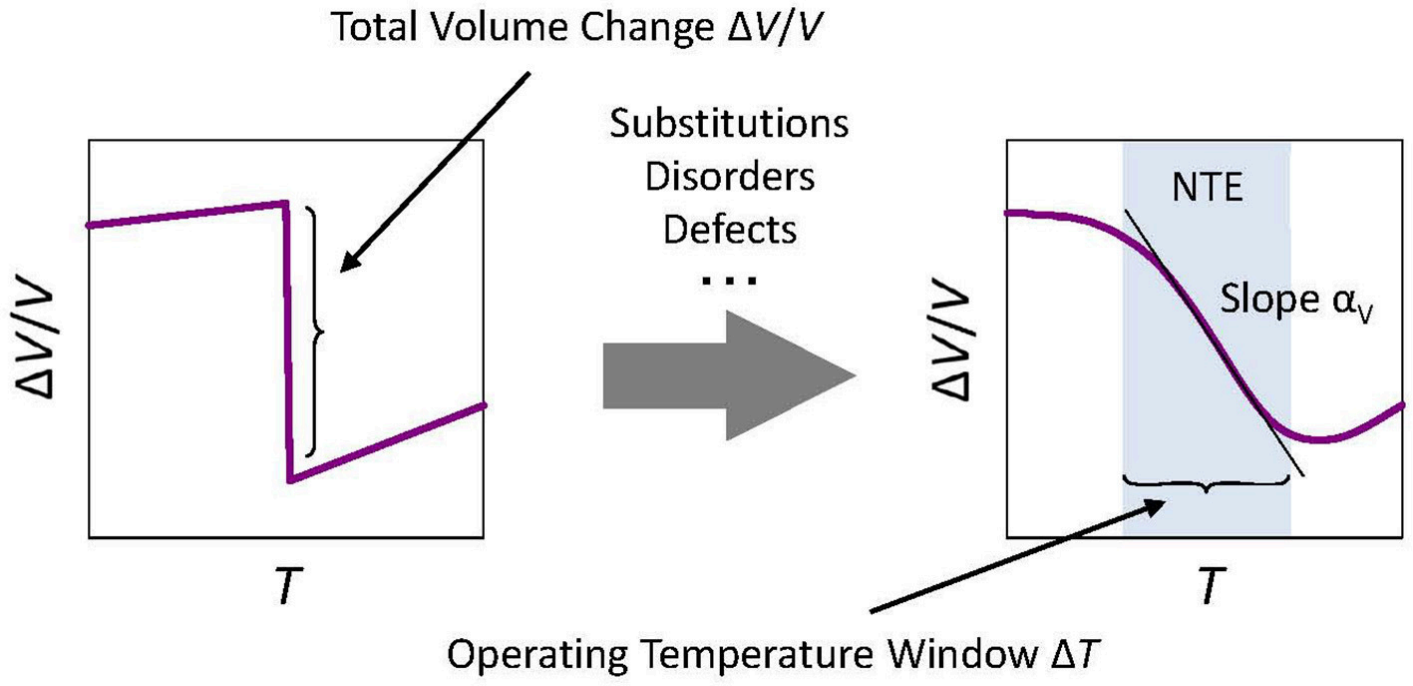
Concept of total volume change Δ*V*/*V* related to negative thermal expansion (NTE). In the case of phase-transition-type NTE materials, “slope” α_V_ (coefficient of *volumetric* thermal expansion) shares a tradeoff relation with the width of the operating-temperature window Δ*T*, roughly described as Δ*V*/*V*~|α_V_|Δ*T*. As an isotropic material, a coefficient of *linear* thermal expansion α_L_ is related to α_V_ as α_V_ = 3α_L_. Therefore, coefficients α_V_ and α_L_ are not intrinsic for such phase-transition-type materials. Instead, the total volume change Δ*V*/*V* is the intrinsic index indicating the potential of NTE.

Various attempts have been made to overcome the shortcomings of the conventional NTE materials presented in the preceding chapter. The current trend of NTE material development is to use the phase transition accompanied by volume contraction upon heating. The effectiveness of this approach was recognized widely by discovery of the giant NTE of α_L_ = −25 × 10^−6^ K^−1^ in antiferromagnetic antiperovskite Mn_3_Zn_1−x_Ge_*x*_N, which uses magnetovolume effects (Takenaka and Takagi, 2005). The giant NTE of manganese nitride strongly influenced subsequent NTE research, leading to the discovery of many NTE materials using magnetic, ferroelectric, charge-transfer, and metal–insulator phase transitions. The phase-transition type NTE concept became dominant in the field (Figure 1). The relation between phase transition and NTE might not be a modern concept because NTE accompanying the magnetic transition (Fe-Pt Invar alloy, Wasserman, 1990) and the ferroelectric transition (PbTiO_3_, Shirane and Hoshino, 1951) has been known for a long time. The novelty of the present method is the modification of the properties of “mother” materials showing a phase transition (in many cases, first-order phase transition), intentionally using chemical approaches such as element substitution (Figure 4).

Specifications of the recently discovered giant NTE materials are presented in Table 1. Phase-transition type materials have an operating temperature that is limited to several tens to 100 K around the phase transition, but they have a negative α_L_ that is several times to 10 times larger than those of conventional type materials. Although a wide operating temperature window Δ*T* is generally suitable for practical applications, limited Δ*T* presents no difficulty in many cases, such as precision equipment that is expected to operate at room temperatures. Bi_0.95_La_0.05_NiO_3_ exhibits large negative α_L_ of −82 × 10^−6^ K^−1^ (Azuma et al., 2015), which is comparable to the magnitude of positive α_L_ of epoxy resin, although the operating-temperature window Δ*T* is not so wide (60 K). Despite that operating temperature window limitation, it is of great importance that it suppresses the thermal expansion of plastics, which has heretofore been difficult.

**Table 1:** Parameters related to *crystallographic* negative thermal expansion for prototypical materials.

**Materials**	**Δ*V*/*V* [%]**	***T*_NTE_ [K]**	**Δ*T* [K]**	**α_L_[ppm/K]^a^**	**Structure^b^**	**Category^c^**	**Method^d^**
β-eucryptite (Gillery and Bush, 1959)	0.15	293–1,073	780	−0.6	Hexagonal	CV	X
ZrW_2_O_8_ (Evans, 1999)	2.7	2–1,443	1,441	−6 ~−9	Cubic	CV	D/N
Cd(CN)_2_·*x*CCl_4_ (Phillips et al., 2008)	2.1	170–375	205	−34	Cubic	CV	X
Mn_3_Ga_0.7_Ge_0.3_N_0.88_C_0.12_ (Takenaka and Takagi, 2005)	0.5	197–319	122	−18	Cubic	MG	D
LaFe_10.5_Co_1.0_Si_1.5_ (Huang et al., 2013)	1.1	240–350	110	−26	Cubic	MG	D
MnCo_0.98_Cr_0.02_Ge (Zhao et al., 2015)	3.2	122–332	210	−52	Orthorhombic	MG	D
0.4PbTiO_3_-0.6BiFeO_3_ (Chen et al., 2006)	2.7	298–923	625	−13	Tetragonal	FE	X
SrCu_3_Fe_4_O_12_ (Yamada et al., 2011)	0.4	180–250	70	−20	Cubic	CT	X
Bi_0.95_La_0.05_NiO_3_ (Azuma et al., 2011)	2.0	320–380	60	−82	Triclinic	CT	D
Sm_2.75_C_60_ (Arvanitidis et al., 2003)	0.8	4–30	26	−100	Orthorhombic	CT	X
Ca_2_Ru_0.9_Mn_0.1_O_4_ (Qi et al., 2012)	0.8	150–400	250	−10	Orthorhombic	MI	X
AgI (Lawn, 1964)	6.0	430	–	–	Hexagonal	Others	X

*Microstructural effects are not considered. Parameters of materials with phase transition accompanied by large volume contraction upon cooling, not broadened, are also listed for comparison*.

a *Averaged value when the material is anisotropic*.

b *For NTE region or lower-temperature, larger-volume phase*.

c *CV, conventional; MG, magnetic transition; FE, ferroelectric transition; CT, charge-transfer transition; MI, metal–insulator transition*.

d *D, dilatometry; N, neutron diffraction; X, X-ray diffraction*.

For phase-transition-type NTE materials, a certain mechanism causes excessive shrinkage that can overcome positive lattice thermal expansion. The mechanisms of prototypical phase-transition-type NTE are described in this chapter. It is noteworthy that the mode of classification, including the conventional type, is not absolute. For example, ferroelectric transition and charge-transfer transition have a common point of charge disproportionation. Other phase transitions such as structural phase transition are often coupled with the magnetic transition.

It is noteworthy that slope α_L_ presents a tradeoff relation with the width of operating-temperature window Δ*T* in the case of phase-transition-type NTE materials (Figure 4). The total volume change Δ*V*/*V* is a value that is peculiar to the substance. It is difficult to increase it artificially. As a result, the wider the operating-temperature window Δ*T* becomes, the smaller the negative slope α_L_ becomes. For isotropic materials, we obtain the simple relation of Δ*V*/*V* = 3α_L_Δ*T*. Therefore, slope α_L_ is not intrinsic for such phase-transition-type materials. Instead, Δ*V*/*V* is the intrinsic and the most important index indicating the potential of NTE. The word “giant” is customarily used in the field, but no clear definition exists. One important turning point was the discovery of ZrW_2_O_8_ in 1996, and a material that surpasses it by the negative coefficient of linear thermal expansion and/or the total volume change related to NTE: a so-called giant NTE material.

### Magnetic transition

**Figure 5:**
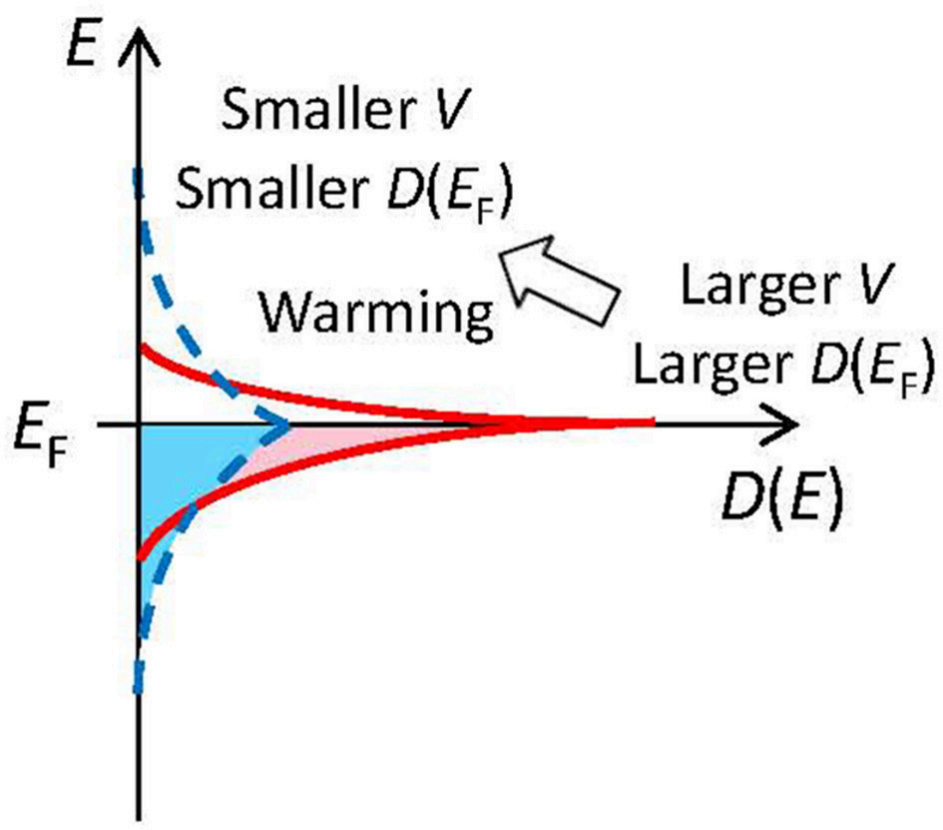
Relation between volume and density of states at the Fermi level *D*(*E*_F_) (schematic). Volume expansion shrinks the electronic band width and therefore enhances *D*(*E*_F_), which is favorable for the magnetic state. It also strengthens the electronic repulsion effects.

The magnetovolume effect is a phenomenon by which the volume changes according to the amplitude of the magnetic moment in itinerant systems. The relative volume change attributable to magnetism is known as the spontaneous volume magnetostriction ω_s_. Generally speaking, less-itinerant electrons are necessary for magnetism. Electronic spins are aligned via the exchange interaction. That exchange interaction does not work well when electrons are itinerant. Volume expansion decreases the overlap of electronic orbitals and makes electrons less itinerant. This effect is explainable by the electronic theory of solids as follows: volume expansion suppresses the bandwidth and condenses the density of states near the Fermi level *E*_F_, which is helpful for magnetism (Figure 5). Therefore, if possible, the system expands the volume to assist magnetism when the magnetic order sets in. This expansion is the magnetovolume effect, which is intimately related with the origin of magnetism in metals. Therefore, it has persisted as a central topic in the field of condensed-matter physics.

The materials classified into this category include 36Ni-Fe alloy (Invar alloy, which has low thermal expansion rather than negative thermal expansion) (Wasserman, 1990), Fe_3_Pt (Sumiyama et al., 1979), YMn_2_ (Nakamura et al., 1988) in the past, and Mn_3_*A*N (where A is a transition metal or semiconducting elements, Takenaka and Takagi, 2005; Hamada and Takenaka, 2011; Wang et al., 2012; Takenaka et al., 2014), La(Fe, Si, Co)_13_ (Fujita et al., 2001; Huang et al., 2013), and MnCo_0.98_Cr_0.02_Ge (Zhao et al., 2015) in recent years. Mass production technology developed in 2012 for manganese nitrides has made them commercially available (Smartec®; Kojundo Chemical Lab. Co. Ltd., Japan, Kawahara et al., 2017).

The low thermal expansion of 36Ni-Fe Invar alloy was reportedly explained by the difference in atomic radius in the early stages of research. The concept of the 2γ model (Weiss, 1963) is that upon heating, the spin configuration of Fe changes gradually from a high-spin state with a larger radius to a low-spin state with a smaller radius. A controversy has long persisted (van Schilfgaarde et al., 1999) between this atomic picture and the itinerant-electron picture described in earlier paragraphs, which emphasizes the contribution from electronic density anomaly near *E*_F_ (Wohlfarth, 1977; Moriya and Usami, 1980). Although details are not included herein, metallic electronic images are regarded as superior in the sense that they can universally explain the magnetovolume effect of various materials, not merely Invar alloys. Nevertheless, differences in atomic radius between high-spin and low-spin states can play an important role in volume change in real materials such as cobalt oxides (Burley et al., 2004). The 2γ model, which still holds importance as a possible mechanism of NTE, is closely related to NTE caused by charge transfer in the sense that NTE is induced by a change in the atomic radius.

Negative thermal expansion is reported also for localized-moment magnets of certain kinds. Actually, NTE is known to appear in the *T* range of several tens of degrees on the high *T* side of the magnetic transition with a compound having a certain spinel or breathing pyrochlore structure (Hemberger et al., 2007; Okamoto et al., 2018; Pokharel et al., 2018). In these materials, NTE disappears below the critical temperature at which the magnetic long-range order is formed. From the localized-moment picture, the magnetovolume effect is attributed to the peculiar volume dependence of the magnetic exchange interaction *J*, ∂*J*/∂ω (ω = Δ*V*/*V*) (Hausch, 1973). The generalized framework extended to the strain derivative of *J* is also discussed (Filippetti and Hill, 2000). The argument based on the Bethe–Slater curve of single *J* discussed previously is too naive to explain the diverse phenomena of real materials. However, if there exist multiple (e.g., ferromagnetic and antiferromagnetic) *J* and competition between them, then the once discarded localized-moment picture might be revived as worthy of consideration.

### Ferroelectric transition

Actually, NTE is widely known to appear in ferroelectric materials with charge disproportionation such as PbTiO_3_ (Shirane and Hoshino, 1951), 0.4PbTiO_3_-0.6BiFeO_3_ (Chen et al., 2006), Sn_2_P_2_S_6_ (Rong et al., 2016), and Pb(Ti,V)O_3_ (Pan et al., 2017). From general consideration of the relation between valence and bond length, the averaged bond length in the charge disproportionate case is greater than the bond length in the charge uniform case (Evans, 1999, Figure 6). Atomic bonds are readily expanded, but they are difficult to shrink. We can regard that fact as representing a kind of anharmonicity of atomic bonds. It is noteworthy that anisotropy is fundamentally important for NTE materials of this type. Phenomenologically, dielectric polarization corresponds to volume change (Chen et al., 2015), similar to magnetic NTE materials in which magnetic moments are intimately related to the volume change, but their physical origin differs greatly.

**Figure 6:**
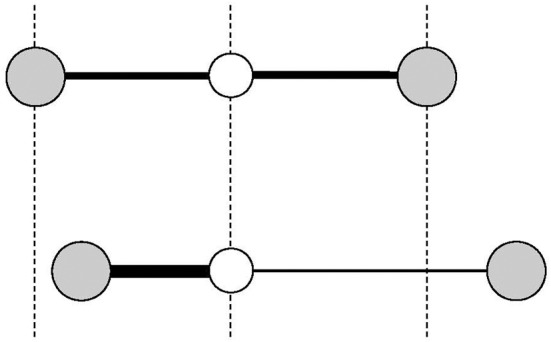
Schematic of negative thermal expansion in a ferroelectric material with charge disproportionation. When charge disproportionation occurs, because the higher-valence interatomic bond length does not shrink as much as the lower-valence one expands **(Bottom)**, the bond length becomes longer than that of the averaged case **(Upper)**.

### Charge-transfer transition

When charge transfer occurs between the constituent elements, atoms with an increasing number of valence electrons become larger. On the other hand, those with a decreasing number of valence electrons become smaller. However, the change in the atomic radius varies according to the atomic species, electronic configuration, etc. In addition, the effect of the atomic radius on the crystal size depends on the atom's position in the crystal structure. If the contribution from atoms with a decreasing number of valence electrons upon heating is greater, then one can expect net NTE.

A famous example of charge-transfer-type NTE materials, bismuth-nickel oxides (Azuma et al., 2011, 2015), is discussed next. When these oxides are warmed, charges are transferred from Ni to Bi. Therefore, the atomic radius of Ni contracts and that of bismuth expands on warming, but the atomic radius of nickel dominates the lattice volume because the Ni–O octahedron dominates the lattice volume. For that reason, the net volumetric NTE occurs in the bismuth-nickel oxide. A similar mechanism functions for NTE of LaCu_3_Fe_4_O_12_ (Yamada et al., 2011).

Negative thermal expansion is also induced by intra-atomic charge transfer that increases the 4*f* electron number with decreasing temperature. For example, Sm_1−x_Y_*x*_S (Jayaraman et al., 1975; Alekseev et al., 2006), Sm_2.75_C_60_ (Arvanitidis et al., 2003), and YbGaGe (Salvador et al., 2003; Sleight, 2003) are known for negative or low thermal expansion by such a mechanism. In the case of Sm, two electronic configurations of (4*f*)^5^(5*d*)^1^ and (4*f*)^6^ compete energetically, but the atomic radius is determined mainly by the 4*f* electron number. Therefore, NTE appears if the electrons are transferred from the 5*d* to the 4*f* orbitals with decreasing temperature.

### Metal–insulator transition

An example in which the effect of electronic repulsion manifests as an apparent physical property is a Mott insulator (Mott, 1990). In transition metal compounds of a certain kind, the metallic state is often lost because of electronic correlations at low temperatures and the system becomes an insulating state. This transition is called a Mott transition. Generally, a Mott insulating state appears at the lower-*T* side. The volume of the low-*T* insulating phase is known to expand upon Mott transition for V_2_O_3_ (1.5%) (McWhan and Remeika, 1970) and NiS (1.8%) (Matoba et al., 1991). The mechanism of volume expansion in the insulating state might resemble a magnetovolume effect in the itinerant-electron magnets (Figure 5). It can be understood that volume expansion suppresses the bandwidth and that it therefore enhances electronic-correlation effects, which stabilizes the low-*T* Mott insulating state. Furthermore, Ca_2_RuO_4_ (Takenaka et al., 2017a) is classified into this category, as explained in the next chapter.

### Other factors

Some materials are also known to have volume of the low-*T* phase which becomes greater than that of the high-*T* phase with the phase transition because of mechanisms other than the four explained above. They are not understood so systematically as the four above, but they are suggestive for the development of new materials. They are therefore explained here. Silver iodide, AgI (Lawn, 1964; Kumar et al., 2006), which is known as a superionic conductor, undergoes a structural phase transition from β and γ phases with larger volume at low temperatures to α phase and smaller volume at high temperatures near 400 K. The volume change is known to reach 5%. The fact that the relation between NTE and ionic conductivity is discussed (Sleight, 1998) also for the famous NTE material β-eucryptite is suggestive of its importance for future development.

Related to a structural phase transition, certain martensitic transformations accompany volume expansion on cooling. For example, MnCoGe is known to expand by 3.9% in the martensitic transformation from high-*T* hexagonal phase to low-*T* orthorhombic phase at around 370 K (Johnson, 1975). Actually, MnCo_0.98_Cr_0.02_Ge, with one of the largest total volume change related to NTE found to date, belongs to the same family and shows the same martensitic transformation simultaneously with the magnetic transition (Zhao et al., 2015). Correlation between magnetism and structure in these materials is an interesting subject that has yet to be explored (Kanomata et al., 1995).

Although the relation between NTE and superconductivity is not well understood, Ca_0.85_La_0.15_Fe_2_As_2_ with the superconducting transition temperature of 43 K exhibits a marked NTE of α_L_ = −16 × 10^−6^ K^−1^ and Δ*V*/*V* = 1.4% at almost all temperatures below 300 K (Robello et al., 2012). Orbital ordering is regarded as involving the Mott transition in Ca_2_RuO_4_ (Mizokawa et al., 2001; Qi et al., 2010) and, although weak, NTE appears just above the Verwey transition (ca. 110 K) in Fe_3_O_4_ (Wright et al., 2000). These results suggest that an orbital-ordering transition might be a universal mechanism of NTE.

### Mechanism of broadening volume change

From the viewpoint of the mechanism of how the sharp volume change accompanying the first-order phase transition becomes a gradual change with temperature, the phase-transition-type NTE materials are broadly divisible into two categories: “phase-separation” type and “second-order-transition” type. In the former category, which includes bismuth-nickel oxides (Azuma et al., 2011, 2015), the system separates into the larger-unit-cell L phase and the smaller-unit-cell S phase, while preserving the first-order phase transition. NTE is induced because the volume fraction of the L phase increases concomitantly with decreasing *T*. In the latter category, which includes magnetic NTE materials such as antiperovskite manganese nitrides (Takenaka and Takagi, 2005; Hamada and Takenaka, 2011; Wang et al., 2012; Takenaka et al., 2014), the phase transition changes from first-order-like to second-order-like. Negative thermal expansion of these materials is characterized by a gradual increase in the unit-cell volume of a single phase with decreasing *T without* phase separation. For manganese nitrides, a detailed neutron diffraction study (Iikubo et al., 2008) demonstrated that the evolution of the amplitude of the magnetic moment is in one-to-one correspondence with the volume increase due to NTE. For the phase-transition-type materials, the mechanism of broadening the volume change is a key issue dominating the operating-temperatures of NTE. To elucidate the broadening mechanism, a certain kind of diffraction study and microscopic observations are required in addition to dilatometry.

## Anisotropic thermal expansion and microstructural effects

**Figure 7:**
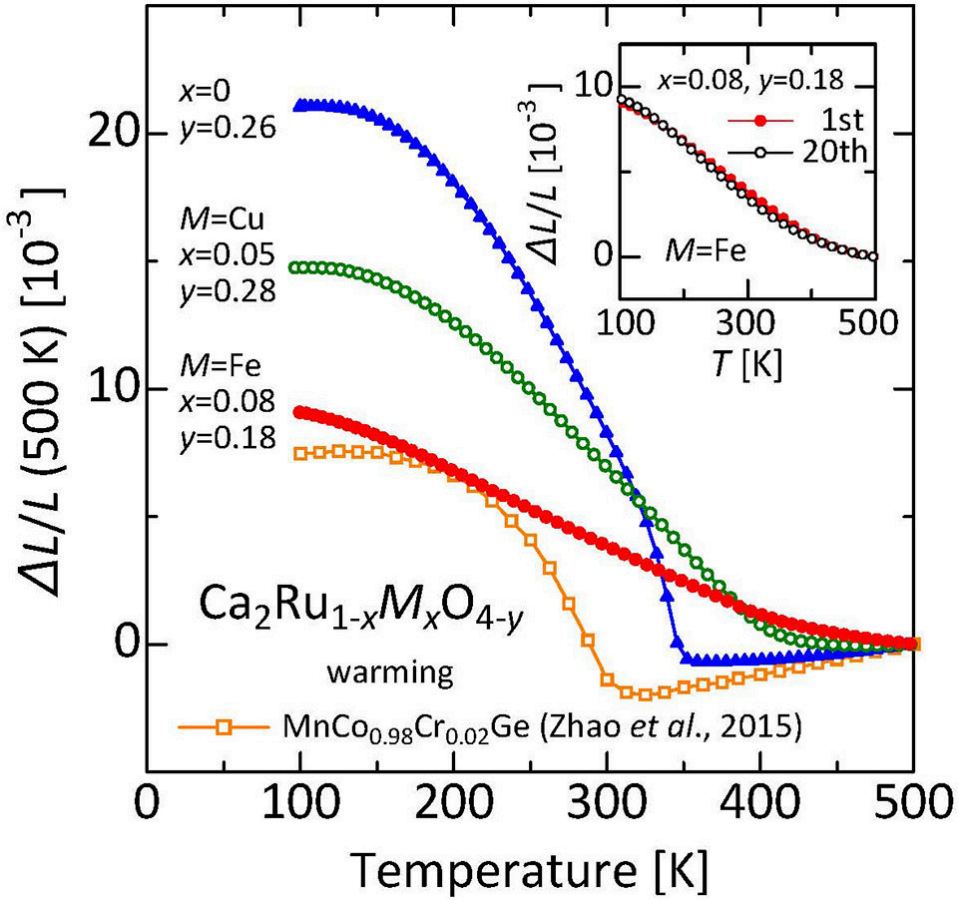
Linear thermal expansion of Ca_2_Ru_1−x_*M*_x_O_4−y_ (*M* = Fe, Cu). Reference temperature: 500 K. Inset: Linear thermal expansion Δ*L/L* of Ca_2_Ru_0.92_Fe_0.08_O_3.82_ before and after 20 iterations of thermal cycling (reference temperature: 500 K). Data related to the warming process were collected using a laser interference dilatometer. Referred from earlier reports (Takenaka et al., 2017a,b). For comparison, data of MnCo_0.98_Cr_0.02_Ge (Zhao et al., 2015) are also shown.

Negative thermal expansion discussed in the former chapters is associated with NTE of the crystallographic unit cell. For these materials, NTE of the unit cell found using a crystallographic technique such as x-ray diffraction and NTE of the bulk measured using a dilatometric technique are fundamentally equal. However, in some cases, the crystallographic NTE and the dilatometric NTE do not coincide. Actually, β-eucryptite, which is a representative practical thermal-expansion compensator, is one example. The colossal NTE measured in the sintered body of layered ruthenium oxide Ca_2_RuO_4_ (Takenaka et al., 2017a) reaffirmed this discrepancy as an important subject in NTE research (Figure 7). The total volume change Δ*V*/*V* related to NTE reaches a maximum of 6.7%, exceeding twice the former maximum (3.2%) obtained with MnCo_0.98_Cr_0.02_Ge (Zhao et al., 2015). Despite the wide operating-temperature window of 135–345 K (Δ*T* = 210 K), α_L_ shows a remarkable size of −115 × 10^−6^ K^−1^ because of this large total volume change.

Originally, Ca_2_RuO_4_ was known for unit-cell volume expansion of about 1% at the onset of high-*T* metallic to low-*T* insulating phase transition at 360 K (Friedt et al., 2001; Qi et al., 2012). Therefore, Ca_2_RuO_4_ is classified as the metal–insulator transition category of NTE described in the preceding chapter. Even in our sample showing a huge bulk NTE, during warming from 150 to 340 K, the *c* axis expands by 4.6%, whereas the *a* axis and the *b* axis shrink respectively by 0.6 and 5.0%. As a result, the unit cell shrinks by 1%. The anisotropic thermal expansion of this unit cell and the fairly large NTE with volume change of Δ*V*/*V* = 1% are regarded as attributable to the Mott transition coupled with electronic orbital ordering, although the details remain to be explored.

**Figure 8:**
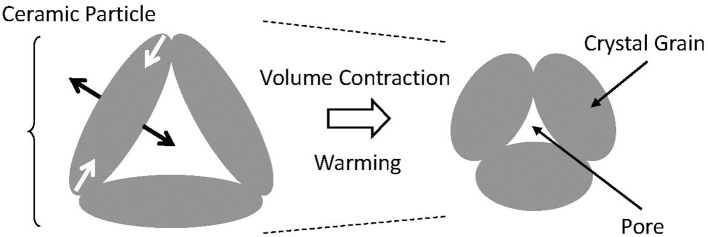
Schematic explanation Δ*L/L* of microstructural effect for bulk negative thermal expansion. The ceramic body consists of crystal grains with anisotropic thermal expansion and pores. When the temperature increases, the crystal grain expands in one direction and contracts in a perpendicular direction. If there exists open space in the direction in which the crystal grain expands, then this ceramic body contracts.

The colossal NTE of Ca_2_RuO_4_ ceramics is proposed as explainable by anisotropic thermal deformation of the crystal grain which fills in the volume of pores that are contained in the sintered body (Figure 8). Such deformation is trivial in the field of ceramics. Moreover, it is not limited to β-eucryptite, for example, also known as MgTi_2_O_5_ (Kuszyk and Bradt, 1973; Ohya et al., 1987; Yamai and Ota, 1993). In most cases, however, it is a “one-time” phenomenon that induces microcracks, which is useless as a practical thermal-expansion compensator. One well-known exception is β-eucryptite, the practical use of which was achieved by overcoming the difficulty of advanced control of microcracks (Pelletant et al., 2012; Benavente et al., 2016). Although the NTE characteristics of β-eucryptite vary according to fabrication conditions, the total volume change of the bulk specimen (1.7%) reaches 10 times that of the unit cell (0.15%); also, α_L_ is −7 × 10^−6^ K^−1^.

The ruthenium oxides are characterized by rather low onset temperatures of anisotropic thermal expansion of the unit cell, which is about 500 K. For many other materials including β-eucryptite and MgTi_2_O_5_, the onset temperature is close to the sintering temperature, typically over 1,300 K. Therefore, the inner stress accumulates greatly during cooling to room temperature. Compared with those, the accumulated inner stresses are expected to be much smaller for the ruthenates. Indeed, the linear thermal expansion Δ*L*(*T*)/*L* of Ca_2_Ru_0.92_Fe_0.08_O_3.82_ is reproducible after 20 repetitions of thermal cycling of 300 K → 500 K → 300 K (inset of Figure 7), which suggests that possible degradation of the characteristics because of the thermal history is avoidable. Recently reported theoretical calculations indicate that the microstructural effects *without* cracks can reproduce the gigantic NTE observed in the ruthenate sintered body (Takezawa et al., 2018). Enhancement of NTE in the ruthenates occurs at a scale of 10 times that of β-eucryptite. Examples for which the material structure is deeply involved in the bulk function of NTE do not exist among recently discovered NTE materials. The concept of microstructural effects provides a strategy for realizing giant NTE. Control of thermal expansion using specific characteristics of materials, particularly in a negative α region, are highly limited in an operating temperature and/or a magnitude of α because of the severe constraints of available materials. To overcome these difficulties, designated structures consisting of two materials having different (positive) thermal expansions and voids are proposed as an artificial materials showing NTE (Takezawa et al., 2015; Wang et al., 2016). The present result is expected to stimulate those activities as a “natural” counterpart of the artificial structures. Structures composed of Structures composed of crystal grains exhibiting anisotropic thermal expansion and voids are expected to be important subjects of future NTE research.

## Composites containing giant NTE materials

Forming a composite using a NTE material as a thermal-expansion compensator is an effective method of controlling the thermal expansion of a material. Many commercial materials exist such as zero-expansion glass used for precision optical equipment and a zero-expansion ceramic material used for semiconductor device manufacturing. Many earlier studies have examined composites containing NTE materials, particularly β-eucryptite (Xue et al., 2010; Borrell et al., 2012) and ZrW_2_O_8_ (Holzer and Dunand, 1999; Matsumoto et al., 2003; Sullivan and Lukehart, 2005; Lind et al., 2011), as thermal expansion compensating fillers. Regarding phase-transition-type giant NTE materials, composites containing Mn_3_*A*N (Ding et al., 2011; Takenaka et al., 2012, 2015; Takenaka and Ichigo, 2014; Lin et al., 2017), BiNi_1−x_Fe_*x*_O_3_ (Nabetani et al., 2015), La(Fe, Si, Co)_13_ (Shan et al., 2015), and Ca_2_Ru_0.92_Fe_0.08_O_3.82_ (Takenaka et al., 2017b) have been reported. Because their chemical reactivity is higher at high temperatures than those of conventional NTE materials, phase-transition-type NTE materials are difficult to use as thermal expansion compensating filler, particularly in cases of metal matrix composites (MMCs). However, powder metallurgy, which enables us to form MMCs at lower temperatures and in a shorter time than when using the conventional method, produced MMCs containing manganese nitrides (Ding et al., 2011; Takenaka et al., 2012, 2015). Some concern arose about difficulties arising from a large thermal expansion difference at the interface between the matrix and the gigantic NTE material. Additional research on the interfaces is expected to be indispensable for the development of industrial materials with high reliability, although this difficulty has not arisen for some composites at present (Takenaka and Ichigo, 2014). Here, plastic matrix composites (PMCs) containing manganese nitrides and ruthenium oxides are discussed.

Generally, the operating temperatures of phase-transition-type NTE materials are not high (ca. 600 K at highest). Therefore, plastics for which the operating temperature range and composite processing temperature are low are indeed the most compatible matrices. In electronic devices, plastics are used in various components such as printed circuit boards, sealing materials, and substrate-chip bonding films. As miniaturization and high integration of integrated circuits progress, problems originating from thermal expansion mismatch between these plastics and inorganic materials such as semiconductors have become more severe. To suppress the large thermal expansion of plastics, conventional thermal expansion compensators such as silica are insufficient. The use of large NTE of phase-transition-type NTE materials is indispensable. Furthermore, control of thermal expansion at a local region at the micrometer level in an integrated circuit, such as underfill of a three-dimensional integrated circuit (3DIC) is strongly demanded (Kino et al., 2017). It is technically important to miniaturize the particle diameter of a giant NTE material to submicrometer size, or less (Lin et al., 2016; Yang et al., 2016).

Before presenting scientific arguments, to summarize briefly how to evaluate the thermal expansion of composites, the author presents discussion of a simple case in which particles of an isotropic reinforcement are dispersed uniformly in an isotropic matrix for analyzing PMCs containing thermal expansion compensating particles. In that case, thermal expansion of the composite falls between the two bounds: The rule of mixture (ROM) and Turner's model (Takenaka, 2012). A bound, ROM, is given, assuming that thermally induced *stress* is uniform throughout a composite, which is equivalent to saying that the matrix and the filler exhibit their own thermal expansion independently. Consequently, the thermal expansion of a composite is given as the volume-weighted sum of contributions from the matrix and the dispersed filler.

(1) $$\begin{array}{l} \alpha_{\text{c}}={\text{v}}_{\text{m}}\alpha_{\text{m}}+{\text{v}}_{\text{t}}\alpha_{\text{t}} \end{array}$$

Another bound, that of Turner's model, is given as the approximation that *strain* is uniform throughout a composite as a result of elastic interactions between the matrix and the filler at the interfaces. It gives the following estimation.

(2) $$\begin{array}{l} \alpha_{\text{c}}=\left({\text{v}}_{\text{m}}{\text{E}}_{\text{m}}\alpha_{\text{m}}+{\text{v}}_{\text{t}}{\text{E}}_{\text{t}}\alpha_{\text{t}}\right)/\left({\text{v}}_{\text{m}}{\text{E}}_{\text{m}}+{\text{v}}_{\text{t}}{\text{E}}_{\text{t}}\right) \end{array}$$

In the equations above, subscripts c, m, and t respectively denote a composite, matrix, and thermal-expansion compensator. Also, *v*_m_ and *v*_t_ respectively represent the volume fractions of the matrix and thermal-expansion compensator; *v*_m_ + *v*_t_ = 1. *E* represents Young's modulus. In Equation (2), the constituent with the larger elastic modulus contributes more to α_c_ beyond the volume fraction. In the case of PMCs containing NTE filler, the relations α_m_ > α_t_ and *E*_m_ < *E*_t_ are always fulfilled. Therefore, Equation (1), ROM, gives the upper bound; Equation (2), Turner's model, gives the lower bound for α_c_.

**Figure 9:**
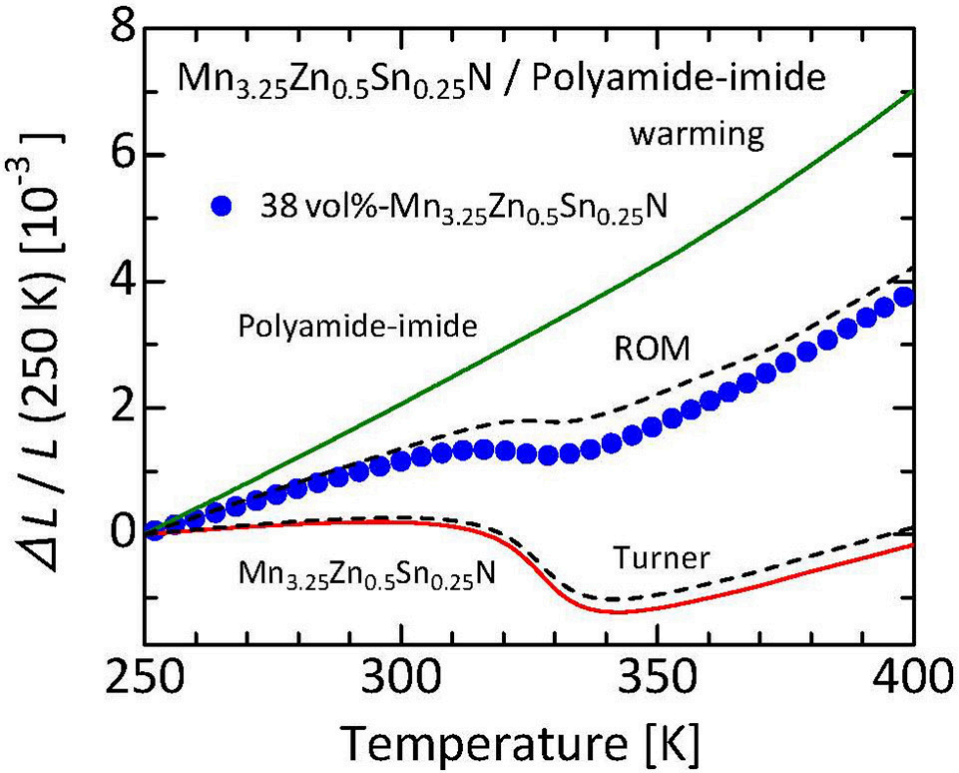
Linear thermal expansion Δ*L/L* of 38 vol%-Mn_3.25_Zn_0.5_Sn_0.25_N/polyamide-imide composite. Reference temperature: 250 K. Data were collected on a warming process using a laser interference dilatometer. Curves calculated by assuming the ROM and Turner's model are also displayed for comparison. Referred from an earlier report (Takenaka and Ichigo, 2014).

Figure 9 displays a plot of the Δ*L*(*T*)/*L* experimental values along with curves calculated assuming ROM and Turner's moles for 38 vol%-Mn_3.25_Zn_0.5_Sn_0.25_N/polyamide-imide composite (Takenaka and Ichigo, 2014). The typical grain size of the nitride filler is 30–50 μm. This nitride thermal expansion compensating filler shows a huge NTE of α_L_ = −45 × 10^−6^ K^−1^ at *T* = 305–340 K, resulting in NTE (α_L_ = −3 × 10^−6^ K^−1^ at *T* = 315–327 K) for the composite. Displacement in Δ*L/L* is less than 160 ppm at *T* = 303–333 K, equivalent to the averaged α_L_ of 5.3 × 10^−6^ K^−1^. The obtained Δ*L*(*T*)/*L* of the composite is between the ROM and Turner's estimates. This result reflects much larger *E* of the nitride filler (200 GPa, Nakamura et al., 2009) than the matrix (5 GPa, Reinecke et al., 1996), demonstrating the excellent thermal expansion compensating capability of the manganese nitride.

The results of bis-phenol epoxy resin matrix composite in which the ruthenium oxide particles are dispersed (Takenaka et al., 2017b) can be discussed next. The filler is Ca_2_Ru_0.92_Fe_0.08_O_3.82_. Although Fe doping somewhat suppresses negative α_L_ to −28 × 10^−6^ K^−1^, the operating temperature window is expanded to 100–500 K. Furthermore, *T*-linearity of Δ*L*(*T*)/*L* is improved. Thermal expansion exhibits almost *T*-linear behavior, even near the lowest temperature used for the present dilatometry measurements. Therefore, NTE apparently continues down to the lower temperature. In that case, the total volume change Δ*V*/*V* might become greater than the present estimate of 2.8% (*T* = 100–500 K). Unfortunately, thermal expansion below 100 K was not measured successfully because of exfoliation of the strain gage. Nevertheless, the composite exhibits low thermal expansion down to 4 K, suggesting NTE of the ruthenate below 100 K.

**Figure 10:**
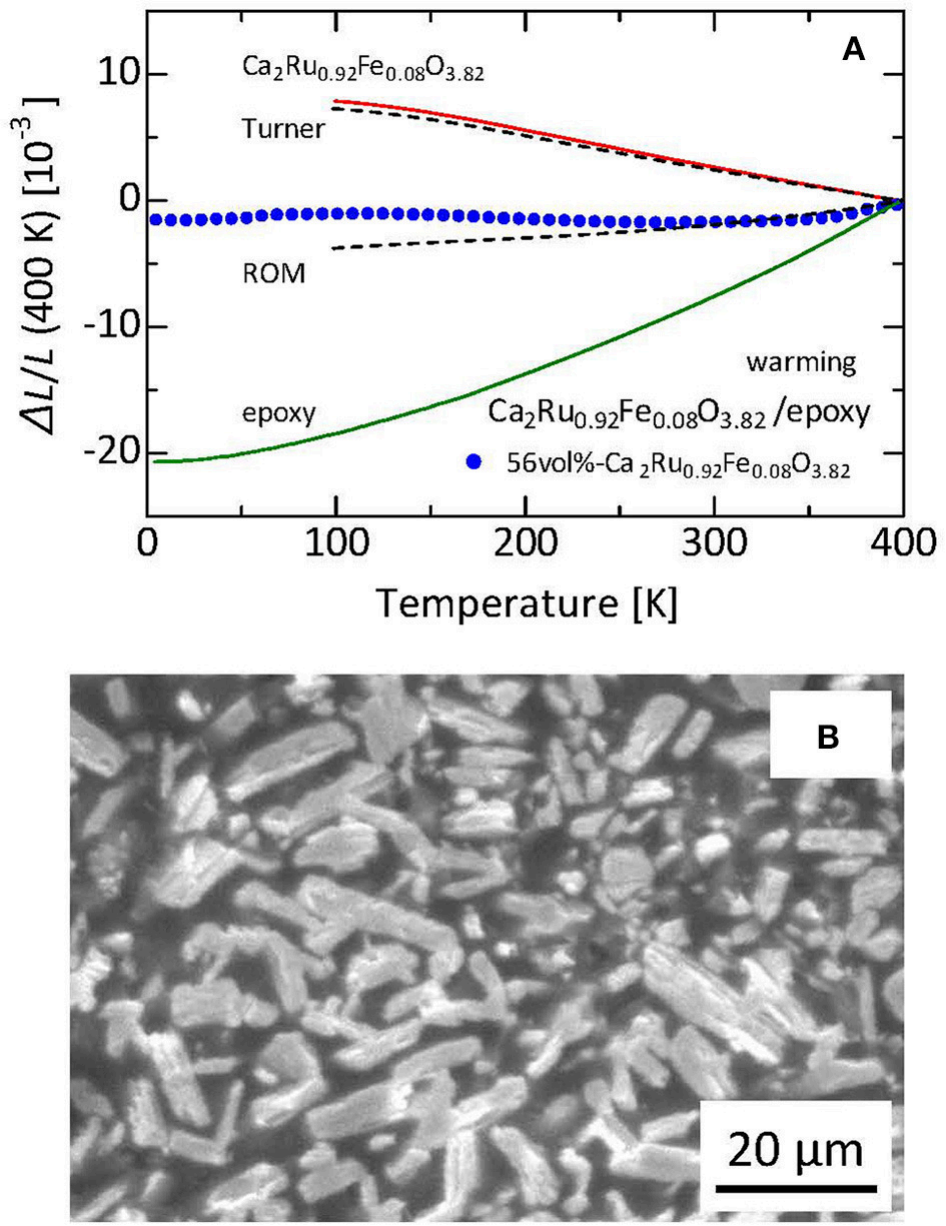
**(A)** Linear thermal expansion (a) and microscopic image **(B)** of 56 vol%-Ca_2_Ru_0.92_Fe_0.08_O_3.82_/epoxy composite. Reference temperature: 400 K. Curves calculated by assuming the ROM and Turner's model are also displayed for comparison. Reproduced from an earlier report (Takenaka et al., 2017b). Copyright (2017) The Japan Society of Applied Physics.

Figure 10A presents a plot of the Δ*L(T)/L* experimental values for the 56 vol%- Ca_2_Ru_0.92_Fe_0.08_O_3.82_/bis-phenol epoxy resin composite (Takenaka et al., 2017b). For the epoxy resin and the composite, Δ*L*(*T*)/*L* was measured down to 5 K using a strain gage. Figure 10B depicts an SEM image of the composite. Dark-gray and light-gray regions respectively represent the epoxy-resin matrix and the ruthenate filler. The ruthenate filler particles, with average size of 20–30 μm, are dispersed uniformly. Each particle is regarded as consisting of smaller crystal grains (ca. 80vol%) and pores (ca. 20vol%). Here, Young's modulus of the epoxy resin is 3.2 GPa (Nabetani et al., 2015). Young's modulus of the ruthenate ceramics particle is estimated as 100 GPa considering the Young's modulus of the related substance Sr_2_RuO_4_ of 160 GPa (Hao et al., 2014) and sample porosity of 0.2 (Wagh et al., 1991). The obtained linear thermal expansion of the composite falls between Equations (1, 2), similar to reports of many earlier studies (Sullivan and Lukehart, 2005; Takenaka and Ichigo, 2014; Nabetani et al., 2015). Therefore, the ruthenium oxide particles dispersed in the composite have sufficient stiffness and negative α_L_. They suppress thermal expansion of the matrix effectively via elastic interactions at the interfaces.

## Concluding remarks

Because of the phase transition accompanied by large volume contraction on heating, giant NTE beyond that of conventional materials has been realized. That achievement has enabled us to compensate the large thermal expansion of plastics, which has been difficult to date, although the operating temperature ranges of the accomplishments have remained limited. The conventional wisdom of materials science, which has held that “negative CTE cannot be so large,” has been rejected. Concepts and technologies related to thermal expansion control are about to change dramatically. One remarkable achievement in recent years is the huge NTE observed for ruthenium oxide sintered bodies. A structure comprising crystal grains exhibiting anisotropic thermal expansion and pores presents fertile ground for the development of new NTE materials. Today, thermal expansion adjustable composites are being fabricated using recently discovered giant NTE materials including the ruthenates.

## Author contributions

The author confirms being the sole contributor of this work and approved it for publication.

### Conflict of interest statement

The author declares that the research was conducted in the absence of any commercial or financial relationships that could be construed as a potential conflict of interest.
